# Effect of Castor and Cashew Nut Shell Oils, Selenium and Vitamin E as Antioxidants on the Health and Meat Stability of Lambs Fed a High-Concentrate Diet

**DOI:** 10.3390/antiox9121298

**Published:** 2020-12-18

**Authors:** Helena Viel Alves Bezerra, Vicente Luiz Macedo Buarque, Lucas Santos Bermudes Silva, Paulo Roberto Pedroso Leme, Ana Maria Centola Vidal, Andréia Cristina Nakashima Vaz, Sarita Bonagurio Gallo, Saulo Luz Silva, Paulo Roberto Leme

**Affiliations:** 1 Department of Animal Science, University of São Paulo, Duque de Caxias Norte av., Pirassununga CEP 13635-900, Brazil; vicentebuarque@usp.br (V.L.M.B.); sbs.lucas@usp.br (L.S.B.S.); prplzoo@usp.br (P.R.P.L.); saritabgallo@usp.br (S.B.G.); sauloluz@usp.br (S.L.S.); 2 Department of Veterinary Medicine, University of São Paulo, Duque de Caxias Norte av., Pirassununga CEP 13635-900, Brazil; anavidal@usp.br (A.M.C.V.); acnvaz@usp.br (A.C.N.V.)

**Keywords:** functional oils, antioxidant, meat shelf life, antimicrobial activity, liver enzyme

## Abstract

Functional oils are known for their compounds with antioxidant, antimicrobial and anti-inflammatory properties, and are used in ruminant nutrition as alternatives to chemicals in order to improve performance. This study aimed to compare the influence of castor and cashew nut shell oils with pure organic selenium (hydroxy-selenomethionine) plus vitamin E, which are known and well-stablished antioxidants, on the performance traits, shelf life and microbial quality of the meat, physiological functions and oxidative stress control of lambs. Thirty-two Dorper x Santa Ines lambs (initial bodyweight of 22.42 ± 3.9 kg and 60 days of age) were submitted to a diet consisting of *Cynodon dactylon* hay (6%) and concentrate (94%). The animals were divided into four treatments: control, without additives; functional oils (FO), 0.50 g/kg DM of castor and cashew nut shell oils; hydroxy-selenomethionine and vitamin E (SeE), 0.50 mg/kg of organic selenium and 100 IU/kg DM of vitamin E; FO plus SeE, at the same doses as the other groups. Blood samples were collected after 1, 30 and 53 days on feed. After 54 days, the lambs were slaughtered and rumen health, carcass and meat traits, shelf life, and microbiological quality were evaluated. There were no differences in performance or carcass traits. A higher muscle and serum Se concentration (*p* < 0.0001), lower lipid peroxidation in meat during display (*p* < 0.0001), and a lower count of psychrotrophic microorganisms on day 5 were observed in the SeE and FO plus SeE groups. The treatments reduced the counts of Enterobacteriaceae, and *Staphylococcus* spp. FO animals showed higher GSH-Px activity on day 30, while the peroxidase activity was higher in FO plus SeE animals (*p* = 0.035). SeE and FO plus SeE animals had lower serum ALT and AST levels. Functional oils improved the microbiological quality of meat. Hydroxy-selenomethionine and vitamin E prevented oxidative stress, lipid peroxidation, and microbial spoilage.

## 1. Introduction

High-concentrate diets are composed of high-energy feeds, mainly corn and other cereals, and although they are not very favorable to ruminants because they need long fiber forage, they are used in intensive production in order to shorten the feeding period, to promote rapid weight gain, to reduce the time to slaughter, and to increase the production of good quality meat [1,2].

However, this nutritional challenge can cause metabolic disorders, such as ruminal acidosis, due to the excessive intake of nonfibrous carbohydrates [3] that can negatively affect plasma metabolites [4]. Furthermore, high-concentrate diets have been widely associated with oxidative stress in ruminants, affecting redox homeostasis in the organism by increasing the metabolic rate [5,6].

Oxidative stress is an imbalance between oxidants and antioxidants that can affect animal production and the general welfare of individuals, and is involved in several pathological conditions that are important for livestock production [7]. Moreover, oxidative stress can damage cell membrane and mitochondrial integrity through lipid peroxidation, which can increase oxidative chain reactions in meat products [8].

Essential oils that improve animal health and productivity have gained increasing attention in livestock production as alternative feed additives [9]. Within this context, castor and cashew nut shell oils are defined as functional oils (FO) because their main components are biosynthesized from fatty acids and their benefits extend beyond their nutritional value [10,11]. Castor and cashew nut shell oils have been used in blends (Essential, Oligo Basics^®^) composed mainly of anacadic and ricinoleic acids, cardol and cardanol. [12]. Cardanol and cardol are related to potential antioxidant activity in vitro and in vivo [13]. Furthermore, the components exhibit antimicrobial activities specially related to the fatty acids in castor oil, and due to the number of terpenoids and phenolic compounds and the interaction between these compounds [11]. Besides that, they are known to improve animal performance, to manipulate ruminal fermentation, to reduce methane production, and to have antitumor activities [11,14].

Selenium and vitamin E are widely used in animal nutrition in order to reduce health disorders induced by oxidative stress, such as sepsis, mastitis, enteritis and metabolic diseases involving dairy ruminants [7]. Thus, since they play an important role in maintaining the redox homeostasis of the organism and the health of animals, it acts in energy redirection for performance improvement [15].

They can also improve the quality of the final product, such as extending the shelf life of meat, since selenium is the main component of glutathione peroxidase enzyme (GSH-Px) [16], the major category of intracellular antioxidant defense, and, as well as vitamin E, act by scavengers of free radicals responsible for lipid peroxidation [17]. It is well-known that their functions extend beyond the antioxidant defense to immunomodulation, especially when dietarily administered, because they can incorporate into cellular membranes [15]. Additionally, there is evidence that pure organic sources of selenium are more bioavailable than inorganic sources, and vitamin E incorporates into cell membranes, fat deposits and other circulating lipoproteins due to its lipophilic character [18,19].

Within this context, this study aimed to evaluate the influence of castor and cashew nut shell oils as a feed additive on performance, rumen health, oxidative stress control and blood parameters, as well as on the microbial quality and lipid stability of the meat of lambs submitted to a high-concentrate diet. The effects of these functional oils were tested in comparison with vitamin E and hydroxy-selenomethionine, a pure organic source of selenium, due to their well-established antioxidant properties.

## 2. Materials and Methods 

### 2.1. Study Site 

This study was carried out at the experimental covered barn associated with the Laboratory of Animal and Meat Quality Assessment of the Department of Animal Science, Faculty of Animal Science and Food Engineering (FZEA), University of São Paulo (USP), Pirassununga, SP, Brazil (21°59′ south latitude and 47°26′ west longitude). All experimental procedures were authorized by the Ethics Committee on Animal Experimentation of FZEA (Approval No. 2,720,110,718).

### 2.2. Animals, Experimental Design and Treatments

The experimental procedures described by Bezerra et al. [17] were followed. Thirty-two just-weaned, male, crossbreed Dorper x Santa Ines lambs (22.42 ± 3.9 kg of initial body weight and 60 days old) were used in order to assess the effect of castor and cashew nut shell oils as antioxidants on oxidative stress control, performance, rumen health, carcass traits, and meat quality, considering that diets with high proportions of concentrate can compromise the redox stability of the organism by boosting the metabolic rate. The lambs were kept in individual pens (2 × 1.25 m) of a covered barn and fed twice daily, adjusted for ad libitum intake, as well as availability of water. The animals were granted 5 days for adaptation, during which they were fed a control diet with no additives, composed of 6% roughage (*Cynodon* sp. hay) and 94% concentrate composed of ground corn, urea, soybean meal and minerals (Table 1).

The National Research Council [3] recommendations for finishing lambs were followed for formulating the diets. The dosage of the blend of castor and cashew nut shell oils was based on manufacturer recommendations (Essential, Oligo Basics^®^). After the end of the 5 days of adaptation, the lambs were designated in a randomized block design with four treatments, with eight replications per treatment: control, with no additives; functional oils (FO), 0.50 g/kg dry matter (DM) of castor and cashew nut shell oils; hydroxy-selenomethionine and vitamin E (SeE), 0.50 mg/kg of selenium in the form of hydroxy-selenomethionine and 100 IU/kg DM of vitamin E (selenium and vitamin E were used in combination for showing synergistic effect [20]); FO plus SeE, at the same doses as the other groups, in order to verify the synergistic effect of the additives administrated in combination. The Se requirement for this lamb category is 0.26 mg/kg DM [3]. The contents of Se per treatment were respectively 0.13, 0.15, 0.49 and 0.45 mg/kg DM. The vitamin E requirement of this sheep category is 5.3 IU per kg of body weight daily [3]. The content of vitamin E for the Control and FO treatments was 11.00 IU/kg DM, and 86.00 IU/kg DM for SeE and FO plus SeE. Estimating according to the requirement, animals with a mean body weight of 30kg who were fed 1.5 kg DM daily consumed 16.5 IU/kg of vitamin E daily in the Control and FO groups and 129 IU/kg of vitamin E daily for the SeE and FO plus SeE groups. The selenium and vitamin E requirements are interdependent [21]. The ingredients were mixed in a horizontal ribbon mixer with a capacity of 500 kg, for 10 min per beat.

The levels of the active compounds of the commercial product (Essential^®^, Oligo Basics) are known and were determined by Near Infrared Spectroscopy (NIRS) technology. Cashew nut shell liquid (40%) and castor oil acid (12%) contain 9% ricinoleic acid, anacardic acid (3-n-pentadecylsalicylic acid), cardanol (3-n-pentadecylphenol; 20%) and cardol (5-n-pentadecylresorcinol; 11%) as the main components [10,22]. The selenium supplement was provided in an organic source as pure hydroxy-selenomethionine (Selisseo^®^ 2% Se, Adisseo SAS France). Vitamin E was provided as α-tocopherol (Microvit^®^ E Promix 50, 500 IU/g, Adisseo SAS France).

**Table 1:** Percent composition of the diets (dry matter basis).

Ingredients (%)	Treatment			
	Control	FO	FO Plus SeE	SeE
Ground corn	77.00	76.95	76.93	76.98
Soybean meal	13.00	13.00	13.00	13.00
Coastcross hay	6.00	6.00	6.00	6.00
Limestone ^1^	2.00	2.00	2.00	2.00
Mineral premix ^2^	0.60	0.60	0.60	0.60
Urea	0.90	0.90	0.90	0.90
Ammonium chloride	0.50	0.50	0.50	0.50
Functional oil ^3^	0.00	0.05	0.05	0.00
Hydroxy-selenomethionine ^4^	-	-	0.0015	0.0015
Vitamin E supplement ^5^	-	-	0.015	0.015
Total	100.00	100.00	100.00	100.00
**Nutrients**				
Metabolizable energy, Mcal/kg ^6^	2.94	2.94	2.94	2.94
Crude protein, %	17.40	17.39	17.39	17.40
Rumen degradable protein, %	10.21	10.20	10.20	10.20
Neutral detergent fiber (NDF), %	16.68	16.67	16.67	16.67
NDF effective, %	6.92	6.91	6.92	6.91
Acid detergent fiber (ADF), %	13.23	11.43	11.07	12.33
N bound to NDF, %	0.26	0.26	0.25	0.31
N bound to ADF, %	0.52	0.52	0.52	0.52
Acid detergent lignin, %	2.48	1.65	1.69	2.77
Calcium, %	0.87	0.87	0.87	0.87
Phosphorus, %	0.42	0.42	0.42	0.42
Selenium, mg/kg	0.13	0.15	0.45	0.49

^1^ Calcium source; ^2^ Coplasal Ovinos^®^ (Coplacana): calcium, 155 g; phosphorus, 83 g; magnesium, 10 g; sulfur, 35 g; sodium, 120 g; copper, 756 mg; zinc, 2800 mg; iodine, 56 mg; cobalt, 44 mg; selenium (sodium selenite), 14 mg; fluorine, 250 mg.; ^3^ Essential, Oligo Basics^®^ (Composed of castor oil acid (12%) and cashew nut shell liquid (40%), cardol (11%), cardanol (20%) and ricinoleic acid(9%)); ^4^ Selisseo^®^ 2% Se, Adisseo; ^5^ Microvit^®^ E Promix 50, (500 IU/g, α-tocopherol), Adisseo; ^6^ Estimated according to Weiss et al. (1992) [23].

### 2.3. Feed Intake and Chemical Analysis

In order to calculate dry matter intake (DMI), the amount of DM available in the diet minus the amount of DM in the leftovers was measured daily. The ratios between weight gain and DMI were used to obtain feed efficiency. The animals were weighed every 14 days in the morning after prior fasting to avoid possible acidosis, in order to measure average daily gain (ADG).

The samples of feed provided and the orts were collected for analysis of dry matter content, non-protein nitrogen, crude protein, neutral detergent fiber and acid detergent fiber, all assessed according to AOAC standards [24]. For total digestible nutrients estimation the methodology proposed by Weiss et al. [23] was followed. The content of Se in the treatments was analyzed by fluorimetry [25]. Table 1 shows the nutrient and chemical compositions of the experimental diets used in this study.

### 2.4. Enzyme Activity and Blood Parameters

Before the beginning of the treatments and after 30 and 53 days on feed, 10 mL blood samples were collected into two Vacutainer^®^ tubes, one with EDTA for the separation of plasma and the other without anticoagulant for the separation of serum, in order to evaluate enzyme activity. 

To analyze oxidative stress, the levels of superoxide dismutase (SOD), glutathione peroxidase (GSH-Px), peroxidases and catalase were measured in plasma instead of serum, because metabolites are better preserved by avoiding the cascade of coagulation and limiting the removal of enzymes from the platelet aggregate, which promotes the better homogenization of the metabolites [17]. Hydrogen peroxide (H_2_O_2_) was measured in serum as described by the specification of the commercial kit. Enzyme activity was measured in plasma or serum using specific commercial kits (BioAssay Systems^®^, CA, USA) for each metabolite and the samples were read in a spectrophotometer (Multiskan GO, Thermo Scientific^®^, Waltham, MA, USA).

Alanine aminotransferase (ALT), aspartate aminotransferase (AST), alkaline phosphatase (ALP), gamma-glutamyl transferase (GGT), and cholesterol were measured in serum using specific commercial kits (Labtest^®^, Lagoa Santa, MG, Brazil) for each metabolite. The samples were read in a spectrophotometer (Mindray BS 120, Guangdong, China).

The specifications of the procedures described by the manufacturer of each kit were followed. The enzyme activities and blood parameters were calculated for each sample from the absorbance values obtained according to manufacturer recommendations.

### 2.5. Slaughter, Sampling and Carcass Traits Assessment

The lambs were carried to the slaughterhouse after 54 days on feed, located 200 m from the experimental barn, in a truck appropriate for sheep. The slaughter procedure followed the guidelines established by the Brazilian legislation (Ministry of Agriculture, Livestock and Food Supply—Decree 9013, March 29, 2017). Briefly, the stunning was performed with a penetrating captive bolt, followed by bleeding through the carotid artery and jugular vein. The carcasses were then skinned, eviscerated, washed, identified, and weighed for the determination of hot carcass weight (HCW).

The temperature and the pH of the carcass were measured after one hour in the longissimus muscle at the height of the 12th rib, with a digital thermometer and pH meter, both supplied with a penetration probe (model HI8314, Hanna Instruments). Then, the carcasses were stored in a cold room (0–2 °C) for 24 h. After, for the determination of cold carcass weight (CCW), the carcasses were again weighed and the temperature and pH were measured at the same location. For the cold carcass yield (CCY) and hot carcass yield (HCY) calculations, the ratios between CCW and HCW were used, respectively, as well as the final body live weight, multiplied by 100.

After, for the measurement of loin eye area, the carcasses were cut into halves and the left half of the carcass was sectioned between the 12th and 13th rib, and a 1 cm^2^ clear plastic grid was used. A millimeter ruler was used in order to measure the subcutaneous fat thickness.

### 2.6. Rumenitis and Rumen Morphology Analysis

After evisceration, the procedures described by Bigham and McManaus [26] were followed. For the determination of rumenitis score, the rumen was separated from the other compartments, opened, and washed. The ruminal papillae were classified visually according to the presence of lesions scored on a scale from 0 to 10, whereby each score indicates 10% of compromised rumen. Any classification above zero was defined as the presence of rumenitis. 

A fragment measuring 3 cm^2^ was collected from the cranial sac of each rumen and stored in flasks with phosphate-buffered saline in order to preserve the integrity of the tissue. Then, the number of papillae/cm^2^ present in the fragment was counted by three persons and the mean of the three counts was considered. After, in order to evaluate the percent papillary area, mean papillary area and the total surface area for absorption per cm^2^ of rumen wall, the fragments and 12 papillae were digitized and analyzed by the UTHSCSA Image Tool [27].

### 2.7. Physical Analysis of Meat

For meat color measurement, the longissimus muscle was sectioned between the 12th and 13th rib for exposure to oxygen for 30 min. Then, color was measured at three different points of the sample with a portable spectrophotometer (model CM2500d, Konica Minolta Brasil) with a D65 illuminant (observer angle of 10° and aperture of 10 mm) previously calibrated with black and white standards and operating in the CIE L*a*b system [28]. The mean value of the three readings was considered as the color value for analysis.

For sheer force analysis, cooking losses and tenderness were evaluated as parameters following the procedures described by the American Meat Science Association [29]. Three steaks from the longissimus muscle with 2.5 cm thickness were excised between the 12th and 13th rib, vacuum packed and frozen for analysis. Prior to analysis, the samples were thawed in a refrigerator at 2–5 °C for 24 h and weighed for the determination of initial weight. Then, each steak had a thermometer inserted into the geometric center and the samples were heated at 170 °C until the internal temperature reached 40 °C in an industrial electrical oven (model F130/L, fornos elétricos Flecha de Ouro Ind. E Com. Ltd.a., Belenzinho, SP, Brazil). The steaks were then turned and cooked until the internal temperature reached 71 °C. Next, the samples were cooled at room temperature (22 °C) and weighed for the determination of final weight. The t difference of initial and final weight of the samples was used in order to calculate the percentage of cooking loss. The samples were then wrapped in plastic film and stored in a refrigerator (4–6 °C) for 24 h. 

After this period, six cylinders 1.27 cm in diameter were excised from each steak in parallel orientation to the muscle fiber and sheared in a texture analyzer (TMS-PRO, Food Technology Corporation, Sterling, VA, USA). The mean value of six cylinders was used in order to obtain the shear force value of each sample and is expressed in Newton (N).

### 2.8. Assessment of Meat Shelf Life Based on Lipid Peroxidation and Microbiological Quality

After 24 h postmortem, and the sectioning of the longissimus muscle between the 12th and 13th ribs, steaks with 1.5 cm thickness were excised, placed on Styrofoam trays separated by treatment, wrapped in PVC film, and randomly allocated on a refrigerated display panel at a temperature of 4 °C and illuminance of 1.000 lux for 5 days. For the analysis of color and lipid peroxidation, samples were collected on days 1, 3 and 5. The samples were analyzed for 2-thiobarbituric acid reactive substances (TBARS) before and after exposure for 1, 3 and 5 days.

For TBARS assessment, 5 g of the exposed steak was triturated and homogenized as described by Vyncke [30]. A standard curve was prepared with five known concentrations of tetraethoxypropane. Absorbance was read in a spectrophotometer (Multiskan GO, Thermo Scientific^®^, Waltham, MA, USA) at 530 and 638 nm. The concentration of malondialdehyde in the samples was obtained with the equation provided by the curve and the results were expressed in µg malondialdehyde/kg tissue.

Microbiological quality was assessed using analytical methods based on the Normative Instruction No. 62 (Brazil, 2003), which regulates the methods for the control of products of animal origin. Twenty-five grams of each exposed steak was collected aseptically and homogenized in 225 mL of sterile 0.1% peptone water with the aid of a stomacher. A standard serial decimal dilution was prepared for each sample exposed for 1, 3 and 5 days.

The presence or absence of *Salmonella* spp. was evaluated after homogenization in peptone water and incubation for 24 h at 37 °C. After incubation, aliquots of 0.1 mL and 1 mL were transferred to Rappaport Vassiliadis Soja broth (incubated at 37 °C/24 h) and Muller–Kauffmann Tetrathionate Novobiocin broth (incubated at 41.5 °C/24 h), respectively. From the tubes, they were lifted on the Xylose Lysine Deoxycholate agar incubated at 37 °C for 24 h. Growth was confirmed by transfer to Triple Sugar Iron agar and incubation at 35 ± 0.5 °C for 18–24 h. 

The counts of psychrotrophic and mesophilic microorganisms were determined using Standard Plate Count agar (Oxoid). The plates were incubated for 10 days at 7 °C for psychrotrophic microorganisms and for 48 at 35 °C for mesophilic microorganisms. Enterobacteriaceae were counted on sterile Petri dishes containing Violet Red Bile Glucose agar (Oxoid); 1 mL of the diluted samples was pipetted onto the plate and homogenized and the plate was incubated for 24 h at 35 °C. For *Staphylococcus* spp. count, a Petri dish containing Baird Parker agar (Difco Laboratories) supplemented with egg yolk–potassium tellurite was inoculated with 0.1 mL of the diluted samples with a Drigalski spatula. After drying, the plates were incubated for 48 h at 35 °C. The colony-forming unit (CFU) mL^−1^ or CFU g^−1^ count in the original sample was obtained by multiplying the number of CFUs by the dilution factor [31].

### 2.9. Statistical Analysis

The data about performance, carcass, meat quality and rumen parameters were analyzed in a randomized block design (initial weight, *n* = 4), with 32 animals allocated to four treatments, totaling eight replicates per treatment. Each animal (pen) was considered an experimental unit. For analysis of the effect of the treatments on the variables studied, the treatment was defined as the fixed effect and the block as the random effect. The means were compared using Tukey’s test (*p* < 0.05). The mathematic model was described:

Y_ij = μ + t_i + b_j + e_ij
(1)

Where:

Y_ij = value of the parcel that received treatment i in block j;

μ = overall mean;

t_i = effect of treatment i, where i = 4;

b_j = effect of treatment j, where j = 4;

e_ij = error associated with the parcel that received the treatment i in the block j.

The results of shelf life analysis (L*, a*, b*, TBARS, and microbiological quality) and the enzyme activity data were analyzed as repeated measures over time considering the treatment, days on display and interaction as fixed effects, while the animal and block were included as random effects. The covariance structures were modeled and the model showing the best fit (composite structure) was used. The results of microbiological analysis were normalized and converted to the base-10 logarithm of the number of CFU plus 1. Microbial counts were analyzed statistically by analysis of variance (ANOVA) and means were compared using Tukey’s test (*p* < 0.05).

The mathematic model used for repeated measures over time was described:

Yijk = μ + αi + D(ik) + β(j) + (αβ) − (ij) + δ_k + E_ijk
(2)

Where:

i = treatment (Control, FO, SeE, FO plus SeE);

j = measures over time;

k = blocks, *n* = 4;

Yijk = subplot value that received the treatments i at time j in block k;

μ = overall mean;

αi = effect of treatment I;

D(ik) = error associated with the treatment i in block k;

β(j) = effect of time j;

(αβ) − (ij) = effect of the interaction between the treatment and the time;

δ_k= effect of block k;

E_ijk = error associated with treatment at time in block.

The rumenitis scores were normalized by the Shapiro–Wilk test to correct for variances and were analyzed using the MIXED procedure for nonparametric analysis. The data were assessed by analysis of variance using the MIXED procedure of the SAS 9.3 software (SAS Institute, Inc., Cary, NC, USA).

## 3. Results

### 3.1. Performance, Carcass, Meat Quality Traits and Rumen Health

The effects of treatments on performance, carcass and meat quality traits are shown on Table 2. There was no effect of the treatments on the performance or carcass traits evaluated. The average daily DMI for lambs was 1.47 kg and the ADG ranged from 317 to 364 g. The CCW ranged from 18.4 to 19.7 kg and the CCY from 47.1 to 47.9%.

No influence of the treatments was observed on most of the meat quality traits evaluated, except for cooking losses in the meat of animals treated with FO plus SeE, which were higher compared to the other treatments (*p* = 0.028; Table 2). The lowest values were observed for the SeE treatment. Shear force remained below 50 N in all treatments. The muscle and serum Se content was higher in animals that received the additional dose of Se (FO plus SeE and SeE treatments) compared to the other treatments (*p* < 0.0001; Table 2).

There was no influence of the treatments on the incidence of rumenitis, absorption area, mean papillary area, papillae number per cm^2^ or total surface area for absorption (Table 3).

### 3.2. Oxidative Stress and Blood Parameters

**Figure 1:**
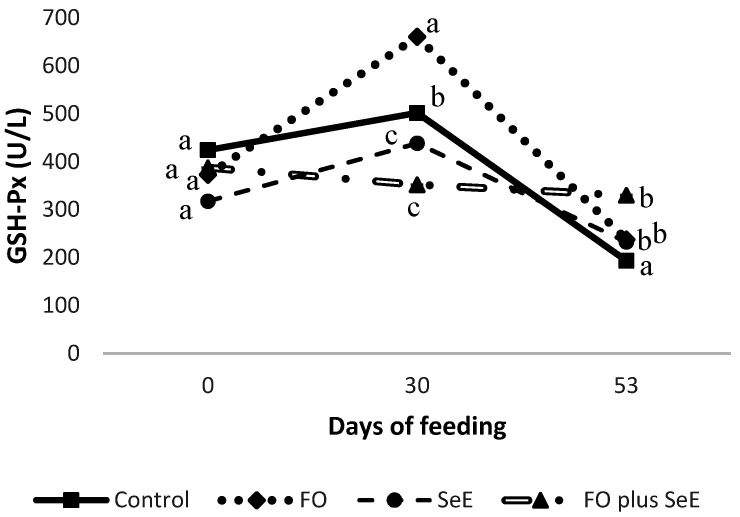
Plasma glutathione peroxidase (GSH-Px) activity obtained for the different treatments according to days of feeding; a–c—treatments in the same time point, followed by different lowercase letters, differ significantly at the 5% level.

No interaction was observed between feedlot time and treatment for plasma SOD, catalase and peroxidases or serum H_2_O_2_, ALT, AST, ALP, GGT and cholesterol (Table 4). However, an interaction between feedlot time and treatment was found for GSH-Px (*p* = 0.006; Figure 1), in which animals that received functional oils (FO) exhibited a higher activity after 30 days on feed; however, no difference compared to the other groups was observed after 53 days, except to the control group. The activity of GSH-Px remained constant throughout the evaluation period in animals that received the combination of functional oils, selenium and vitamin E (FO plus SeE).

The treatments influenced peroxidase levels, in which animals that received FO plus SeE showed higher enzyme activity (*p* = 0.035; Table 4).

Regarding the other blood metabolites, animals treated with FO plus SeE and SeE alone exhibited lower serum activity of ALT (*p* = 0.06) and AST (*p* = 0.033), respectively (Table 4).

### 3.3. Meat Shelf Life, Lipid Peroxidation (TBARS) and Microbiological Quality

There was no significant interaction between treatment and days on display for the a* or b* color parameter, and the treatments exerted no effect on these parameters. The lowest L* values were observed for the meat of animals from control the group (*p* = 0.026; Table 5).

The interaction between treatment and days on display did not affect TBARS values. However, an effect of treatment was observed, in which the meat of the animals that received the additional dose of Se (FO plus SeE and SeE) exhibited the lowest TBARS levels (*p* = 0.001; Table 5).

**Figure 2:**
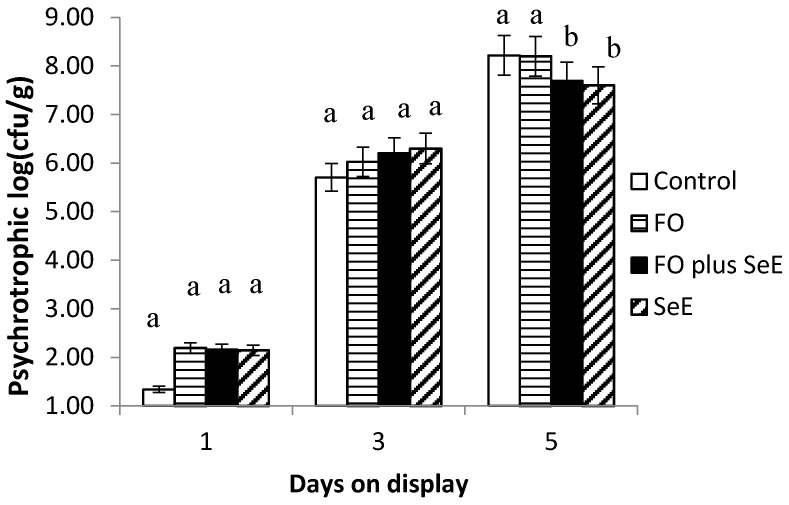
Mean log counts of psychrotrophic microorganisms obtained for the different treatments according to days on display; a, b—treatments in the same time point, followed by different lowercase letters, differ significantly at the 5% level.

Regarding the microbiological quality of meat, an interaction between treatment and days on display was observed for psychrotrophic microorganism count (Figure 2), which was lower after 5 days on display in the FO plus SeE and SeE treatments.

**Figure 3:**
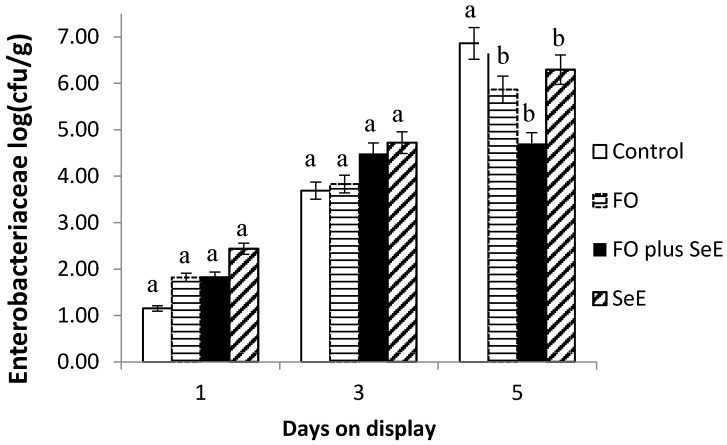
Mean log counts of bacteria of the family Enterobacteriaceae obtained for the different treatments according to days on display; a, b—treatments in the same time point, followed by different lowercase letters, differ significantly at the 5% level.

**Figure 4:**
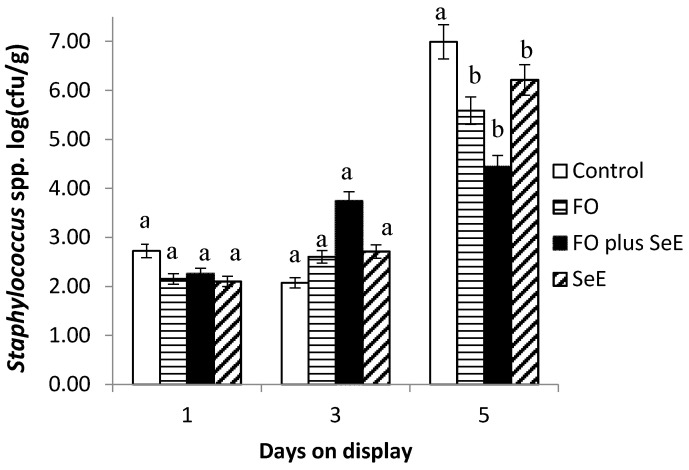
Mean log counts of *Staphylococcus* spp. obtained for the different treatments according to days on display; a, b—treatments in the same time point, followed by different lowercase letters, differ significantly at the 5% level.

Furthermore, an interaction between treatment and days on display was observed for *Enterobacteriaceae* (Figure 3) and *Staphylococcus* spp. (Figure 4). As expected, no difference between treatments was found in the baseline assessment (day 1) and on day 3. After this period (day 5), the meat of animals from all treatments, except for the control, had the lowest CFU counts. The lowest counts were observed for the two treatments containing FO (FO and FO plus SeE) and for the SeE treatment throughout the period of simulated display.

## 4. Discussion

The treatments had no effect on the performance traits evaluated. Daily DMI remained in the recommended range by the NRC [3], from 1.0 to 1.5 kg, as did the ADG (from 317 to 364 g) and CCY (from 47.1 to 47.9%). Similar values have been reported by Bezerra et al. [17] who fed Dorper x Santa Inês lambs with comparable high-concentrate diets with four treatments (control, no additives; 15 mg/kg DM of monensin; 12.5 g/DM of a purified lignin; and 0.33 mg/kg of selenium combined with 100 IU/kg DM of vitamin E) and reported an ADG of 324 to 339 g and a CCY of 46 to 48%.

Diets with high proportions of concentrate contain higher levels of metabolizable energy, which result in more efficient utilization of net energy since the energy requirement for maintenance is diluted, redirecting a portion of the energy to the deposition of tissue in the form of protein or fat [3]. Therefore, the high ADG found in the present study and the lack of influence of the treatments on the performance and carcass traits might be related to the high proportion of concentrate in the diet, which possibly standardized the growth rate. No synergistic relationship between functional oils and selenium plus vitamin E was verified.

The treatments with an additional dose of Se (FO plus SeE and SeE) influenced the muscle and serum Se content of lambs. In the present study, hydroxy-selenomethionine was used as a pure organic source of Se. It is known that selenomethionine increases the bioavailability of Se supplements since the mechanism of absorption of both methionine and selenomethionine is the same, and selenomethionine is nonspecifically incorporated into body proteins, replacing standard methionine [32]. These results agree with Paiva et al. [33], who supplemented lambs with different Se sources and levels and found a higher Se content in the muscle of lambs supplemented with 0.8 mg/kg DM of selenomethionine. However, the serum Se content was higher in all treatments compared to control, regardless of the source. In several countries, the Se requirements for humans (55 μg/d) are generally not met [34]. About three billion people worldwide have Se deficiency because its content in dietary supplements for animals is very variable worldwide [35]. Since selenium is incorporated into selenoproteins with a range of effects in human health, that vary from antioxidant defense to active thyroid hormone, selenium deficiency can cause many impacts and diseases [36]. High selenium status is associated with a low overall mortality of all-cause cardiovascular diseases and cancer [37]. It is important for immune responses [38], and maintaining brain tissue [39] and thyroid function [40]. Therefore, meat may be an excellent source of Se [41] since higher levels are observed in the meat of animals supplemented with organic sources of Se.

Regarding meat quality traits, higher cooking losses were observed in the meat of animals that received FO plus SeE and lower values were found in animals fed SeE. It is known that cooking loss is related to the water-holding capacity (WHC) of meat. The latter parameter is of great importance for the meat industry because it affects sensory and economic attributes such as meat color, tenderness, texture, and juiciness. A higher cooking loss is directly related to a lower WHC and lower juiciness [42,43]. The determinants of WHC in meat are postmortem anaerobic muscle glycolysis, the rate and extent of pH fall, proteolysis, and even protein oxidation. Differences in the antioxidant status of the animal or muscles may affect proteolysis and, consequently, meat quality traits such as tenderness and WHC [44,45]. Calvo et al. [46] supplemented pigs with different sources of selenium (organic Se-enriched yeast and inorganic sodium selenite) and the combination of Se and vitamin E, and verified that the organic source of Se improved WHC. Additionally, the combined administration of sodium selenite and vitamin E (0.2 mg/kg and 100 mg/kg, respectively) decreased myofibrilar protein hydrolysis/oxidation, improving WHC. Those results corroborate the present study, in which SeE treatment possibly influenced the antioxidant status of muscles, contributing positively to WHC and consequently to the lower cooking loss.

Despite the influence of the treatments on cooking losses, no effect on the physicochemical characteristics of meat, such as tenderness or pH, was observed. However, the meat of animals fed FO plus SeE exhibited the highest cooking losses. This change may have been observed due to the influence of anacardic acid present in the FO, since this can induce the production of free radicals [47], unbalancing the antioxidant status of the muscle of animals treated with FO plus SeE. Additionally, this treatment increased plasma peroxidase levels as an antioxidant defense against free radical production induced by anacardic acid, as discussed below, indicating the imbalance between oxidants and antioxidants.

Higher GSH-Px activity was observed in animals treated with FO on day 30. It is known that selenium is an essential constituent of GSH-px [16], and a higher activity of GSH-Px was expected for the groups treated with an additional dosage of selenium (FO plus SeE and SeE). Although organic sources of selenium are more bioavailable [32], the synthesis of selenoenzymes is quicker from an inorganic source, because this form can be rapidly biotransformed into selenocisteyne, the principal amino acid precursor of selenoenzymes [33,48], when compared to the organic selenium form, which can explain the lack of influence of selenium supplementation on GSH-Px activity. However, GSH-Px can also be an indicator of oxidative stress and of antioxidant capacity [49].

Hollands et al. [47] investigated the effects of anacardic acid on the functions of human neutrophils, and found that, despite its in vitro antioxidant properties [50], this natural product stimulates the production of reactive oxygen species (ROS) and neutrophil extracellular traps. Studies suggest that anacardic acid may stimulate cellular superoxide production by inhibiting the small ubiquitin-like modifier (SUMO) modification of NADPH oxidase [51].

Given the importance of ROS and cellular superoxide production for the redox homeostasis of the organism, enzymatic antioxidants have been established as the main type of intracellular antioxidant defense, including GSH-Px [5]. Tüzün et al. [49] investigated the plasma levels of GSH-Px in patients with inflammatory bowel disease compared to a healthy group and found that the plasma levels of GSH-Px were significantly higher in the patients. They concluded that the higher GSH-Px was a response of the oxidative stress induced by free radicals that increased due to the inflammatory bowel disease and, in spite of this, the patients had an antioxidant capacity against free radicals. In the present study, the increase in the activity of GSH-Px in animals fed FO possibly indicates a response to the cellular superoxide production induced by FO, acting as a free radical scavenger, showing the antioxidant capacity of the animals who were fed FO as the main form of intracellular antioxidant defense.

Selenium is the main component of GSH-Px, which is in the peroxidases group. Vitamin E (α-tocopherol) works as a hydrogen atoms donor to cell membranes, which reduces the propagation of the peroxidation chain reaction in lipid membranes [16,52]. Thus, the additional doses of selenium and vitamin E worked as potential antioxidants, attenuating the imbalance between oxidants and antioxidants and protecting against oxidative damage, even in animals treated with combined doses of FO plus SeE. Indeed, animals treated with FO plus SeE showed increased peroxidases levels. According to Pütter [53], peroxidases are a group of oxidoreductases enzymes that work on the reduction of H_2_O_2_ to H_2_O and detoxify free radicals. When elevated H_2_O_2_ levels are detected, an increase in the activity of peroxidases would be the first response of the organism. Here, selenium and vitamin E would act as scavengers of the radicals produced by FO when both additives are combined in the diet, showing an effect against the oxidative stress induced by FO.

Moreover, a lower activity of ALT and AST was observed in animals treated with SeE alone and in combination with FO. According to Pratt and Kaplan [54], these enzymes are biomarkers of hepatocellular injury and increase when the liver cell membrane is damaged. Assuming that high-concentrate diets affect the organism’s redox homeostasis by increasing the metabolic rate, the liver is the vital organ that is more frequently attacked by the ROS generated, since it is the principal detoxifying organ that has a metabolizing role, and due to its high fatty layer. The active generation of ROS can cause liver damage [55].

The lipophilic nature of vitamin E allows its incorporation into cell membranes, fat deposits and other circulating lipoproteins. This vitamin has been used to treat inflammatory diseases because of its antioxidant effect. In addition, selenium plays an important role in the normal processes of the body, including the immune system [19,56]. The decreased activity of ALT and AST compared to the other treatments provides clear evidence that the dietary administration of selenium and vitamin E exerted protective effects on the liver. Despite the fact that animals treated with FO plus SeE showed a lower activity of ALT and AST, no influence of FO alone was observed on these enzyme activities. Toyomizu et al. [57] and Braz et al. [58] found no influence of anacardic acid or the liver enzyme of rats, suggesting no hepatic dysfunction induced by FO.

With respect to the shelf life of meat, the maintenance of the sensory characteristics as well as microbial quality and lipid stability, which are correlated with food security, flavor, color and odor, is an important issue for the meat industry. Dietary antioxidants can be absorbed and can be effective against lipid peroxidation because of their uniform incorporation into the cell membrane [59]. During the simulated display, the SeE and FO plus SeE treatments provided the lowest malondialdehyde levels in TBARS analysis compared to the other treatments. Calvo et al. [46] observed the lowest values of TBARS in the meat after seven days of storage of pork treated with combined sodium selenite and vitamin E (α-tocopheryl-acetate), at 0.2 mg/kg and 100 mg/kg, respectively. Rey et al. [60] observed reduced TBARS values in the meat of pork treated with vitamin E (α-tocopheryl-acetate – 100 mg/kg) and sodium selenite (0.26 mg/kg) alone or in combination with oleuropein extract. In the present study, selenium and vitamin E were effective in maintaining low lipid peroxidation levels throughout exposure due to the lipophilic nature of vitamin E and its mechanism of action by hydrogen donation, in combination with Se.

Despite the fact that FOs are known for their antioxidant activity, in the present study, no influence of FO was observed in preventing the chain propagation of lipid peroxidation in meat. Braz et al. [58] found no difference in lipid peroxidation in the blood serum, liver, magnum and uterus of laying hens fed with cashew nut shell liquid, although lower lipid peroxidation was found in the birds’ ovaries. Ferket et al. [61] found reduced values of TBARS in the meat of turkeys treated with cashew nut shell liquid and castor oil (Essential: Oligo Basics, 0.15%) after seven days of storage. Despite this positive result, it is worth mentioning the differences in dosage and species evaluated.

In addition to promoting lipid stability, the SeE and FO plus SeE treatments promoted the lowest counts of psychrotrophic microorganisms on day 5 of simulated display. These microorganisms, which grow at refrigeration temperatures [62], are indicators of the hygienic condition of foods and are responsible for food spoilage and alterations in sensory characteristics. Microbial growth and metabolism in meat are highly affected by the redox potential, which is determined by the pH, gaseous atmosphere and presence of reductants [63]. The present results suggest that dietary supplementation with selenium and vitamin E affects redox regulation by removing free radicals, reducing the redox potential in muscle tissue so that the medium becomes unsuitable for the growth of aerobic psychrotrophic microorganisms. So far, selenium and vitamin E have been reported to enhance antibiotic activity via an indirect mechanism through the improvement of immune functions [64,65]. No influence of FO alone was observed on log counts for psychrotrophics, suggesting no antimicrobial action against this type of microorganism and no effect on the redox potential in muscle tissue.

Additionally, the regulatory effects on the potential redox of selenium and vitamin E by removing free radicals of the substrate indicated lower counts of bacteria of the family Enterobacteriaceae and *Staphylococcus* spp. Besides that, the meat of lambs treated with FO and FO plus SeE also showed the lowest counts of bacteria of the family Enterobacteriaceae and *Staphylococcus* spp. These microorganisms originate from the skin and gastrointestinal tract of the animals and can contaminate the carcasses if not removed carefully during slaughter [66]. Essential oils are known to interfere with the gastrointestinal microbiota and are generally effective against Gram-positive bacteria such as *Listeria monocytogenes*, *Salmonella typhimurium*, *Escherichia coli* O157:H7, *Shigella dysenteriae*, *Bacillus cereus* and *Staphylococcus aureus* [67]. Furthermore, Moraes et al. [68] found that FO are effective against Gram-positive bacteria such as *Staphylococcus aureus* in the intestinal microbiota of broiler chickens. The reduction in bacteria such as Enterobacteriaceae by FO reduces the risk of carcass contamination and consequent meat spoilage.

As expected, no difference between treatments and CFU counts for any microorganism evaluated was found in the baseline assessment (day 1) and at day 3 of exposure on display. This was expected since all procedures followed the sanitation practices in order to avoid cross-contamination and spreading microorganisms among meat. Additionally, bacterial growth can be explained by a growth curve [69] represented by an initial phase, or lag phase, in which the bacteria adapt to growing conditions and synthesize RNA, enzymes and other molecules. When the adaptation is complete, the microorganisms start an exponential phase of growth, which is very rapidly. The treatments showed effectivity against bacterial growth on meat at day 5 on display, time which represents the exponential growth of bacteria. Also, the treatments showed an effect in extending the quality of the meat on time of exposure on display.

## 5. Conclusions

Under the conditions of this study, neither castor and cashew nut shell oils nor pure hydroxy-selenomethionine and vitamin E affected the performance traits evaluated. However, these functional oils effectively inhibited the growth of Enterobacteriaceae and *Staphylococcus* spp. in meat, although they may compromise the redox homeostasis of the organism. Pure hydroxy-selenomethionine and vitamin E exhibited effective antioxidant activity against oxidative stress and lipid peroxidation, and also provided a secondary mechanism protecting against microbial spoilage by psychrotrophic microorganisms, extending the shelf life of meat and promoting liver health in lambs fed a high-concentrate diet.

For now, the dietary administration in combined form of functional oils, selenium and vitamin E should be considered, since castor and cashew nut shell oils are known for preventing metabolic disorders induced by high-concentrate diets in ruminants, and for improving performance. Besides that, in this study, they improved the microbial quality of meat, although the dietary administration of functional oils for ruminants needs to be associated with a good nutritional management and good hygiene practices when slaughtering and processing meat products. Despite that, more studies in vivo need to be carried out in order to investigate their effects on oxidative stress, meat quality and lipid stability parameters.

## Figures and Tables

**Table 2:** Performance, carcass and meat quality traits (means ± s.d.) of Dorper x Santa Ines lambs fed a high-concentrate diet with different antioxidants.

Traits	Treatment				*p*-Value
	Control	FO	FO Plus SeE	SeE	
Initial BW, kg	22.26 ± 2.41	22.32 ± 2.50	22.79 ± 1.97	22.32 ± 2.18	0.9621
Final BW, kg	40.21 ± 2.06	38.79 ± 3.32	41.71 ± 3.24	39.88 ± 3.04	0.234
ADG, kg	0.35 ± 0.05	0.32 ± 0.03	0.36 ± 0.04	0.34 ± 0.06	0.359
DM intake, kg/day	1.49 ± 0.09	1.50 ± 0.05	1.46 ± 0.22	1.41 ± 0.20	0.817
Feed efficiency, g/kg DMI	0.23 ± 0.04	0.21 ± 0.03	0.25± 0.02	0.24± 0.04	0.229
Hot carcass weight, kg	19.46 ± 0.99	18.94 ± 1.97	20.28 ± 0.04	19.58 ± 1.07	0.252
Hot carcass yield, %	48.42 ± 1.46	48.78 ± 1.67	48.61 ± 1.07	49.16 ± 1.67	0.791
pH 1 h	6.45 ± 0.19	6.58 ± 0.14	6.63 ± 0.12	6.49 ± 0.20	0.144
Temperature 1 h, °C	30.11 ± 2.82	29.35 ± 1.34	29.59 ± 2.51	29.49 ± 1.81	0.915
Cold carcass weight, kg	18.94 ± 1.05	18.41 ± 2.06	19.71 ± 1.59	19.08 ± 1.06	0.298
Cold carcass yield, %	47.10 ± 1.48	47.39 ± 1.79	47.25 ± 1.14	47.91 ± 1.61	0.741
pH 24 h	5.87 ± 0.42	5.82 ± 0.34	5.68 ± 0.06	5.69 ± 0.09	0.423
Temperature 24 h, °C	11.59 ± 3.25	12.99 ± 3.49	12.98 ± 2.48	13.26± 2.95	0.699
Loin eye area, cm^2^	28.00 ± 5.13	30.25 ± 7.18	30.38 ± 6.05	32.38 ± 4.65	0.560
Subcutaneous fat thickness, mm	7.75 ± 4.40	5.75 ± 2.18	7.88 ± 2.35	6.50 ± 2.56	0.447
Cooking loss, %	24.58 ± 4.30 ab	26.66 ± 3.34 ab	29.91 ± 4.32 a	23.81 ± 4.35 b	0.028
Shear force, N	39.66 ± 11.77	42.34 ± 12.56	50.85 ± 8.41	47.33 ± 11.40	0.231
Muscle selenium, mg/kg	0.07 ± 0.01 b	0.05 ± 0.007 b	0.10 ± 0.01 a	0.11 ± 0.01 a	<0.0001
Serum selenium, mg/kg	0.11 ± 0.001 b	0.11 ± 0.01 b	0.15 ± 0.009 a	0.13 ± 0.01 a	<0.0001

BW, body weight; ADG, average daily gain; DM, dry matter; DMI, dry matter intake. Means in the same row followed by different lowercase letters differ significantly at the 5% level. Control: without additives; FO: functional oils, 0.50 g/kg DM of castor and cashew nut shell oils; SeE: hydroxy-selenomethionine and vitamin E, 0.50 mg/kg of selenium and 100 IU/kg DM of vitamin E; FO plus SeE, at the same doses as the other groups.

**Table 3:** Rumenitis incidence and ruminal morphology parameters (means ± s.d.) of Dorper x Santa Ines lambs fed a high-concentrate diet with different antioxidants.

Parameters	Treatment				
	Control	FO	FO Plus SeE	SeE	*p*-Value
Rumenitis score	0.88 ± 0.64	1.63 ± 1.84	1.13 ± 0.99	1.50 ± 1.06	0.551
Papillary area, cm^2^	0.24 ± 0.04	0.26 ± 0.08	0.31 ± 0.09	0.30 ± 0.10	0.378
Absorption surface, cm^2^ of wall	14.68 ± 2.89	16.44 ± 5.45	15.88 ± 4.31	15.13 ± 5.83	0.899
Absorption surface, %	93.67 ± 1.91	94.04 ± 2.15	93.88 ± 1.96	93.18 ± 2.71	0.890
Papilla number/cm^2^ of wall	59.15 ± 18.38	59.41 ± 9.72	49.50 ± 10.24	47.51 ± 12.49	0.172

Control: without additives; FO: functional oils, 0.50 g/kg DM of castor and cashew nut shell oils; SeE: hydroxy-selenomethionine and vitamin E, 0.50 mg/kg of selenium and 100 IU/kg DM of vitamin E; FO plus SeE, at the same doses as the other groups.

**Table 4:** Plasma and serum oxidative stress and blood parameters (means ± s.d.) of lambs fed a high-concentrate diet with different antioxidants.

Parameters	Treatment				Days			*p*-Value		
	Control	FO	FO Plus SeE	SeE	0	30	53	Treatment	Time	Treatment * Time
SOD	0.261 ± 0.42	0.278 ± 0.34	0.375 ± 0.44	0.438 ± 0.47	0.410 ± 0.29	0.407 ± 0.37	0.197 ± 0.38	0.412	0.072	0.369
GSH-Px	373.12 ± 213	423.62 ± 235	356.57 ± 113	329.58 ± 150	375.56 ± 102 b	488.14 ±149 a	248.46 ± 132 c	0.157	<0.0001	0.006
Catalase	2.18 ± 2.26	2.34 ± 1.44	1.64 ± 2.79	2.46 ± 1.25	2.32 ±0.96	1.71 ±1.75	2.44 ± 1.49	0.531	0.353	0.852
Peroxidases	3.58 ± 1.37 b	4.57 ± 1.27 ab	8.11 ± 1.40 a	2.65 ± 1.30 b	5.56 ± 1.17 a	2.23 ± 1.16 b	6.40 ± 1.44 a	0.035	0.031	0.539
H_2_O_2_	932.56 ± 478	896.10± 181	891.83 ± 181	906.45 ± 247	988.38 ± 375	894.08 ± 261	837.74 ± 236	0.972	0.146	0.273
ALT	12.00 ± 4.81 a	14.24 ± 6.54 a	9.08 ± 5.74 b	9.71 ± 3.93 b	13.00 ± 4.93	10.46 ± 5.07	10.31 ± 5.16	0.006	0.095	0.935
AST	112.62 ± 32.54 a	108.24 ± 35.83 a	92.75 ± 14.58 b	98.00 ± 25.78 b	84.33 ± 10.34 c	113.13 ± 20.14 ab	111.25 ± 21.63 b	0.033	<0.0001	0.641
ALP	593.24 ± 208	608.79 ± 250	609.75 ± 165	600.29 ± 216	651.91 ± 187	605.49 ± 209	551.66 ± 209	0.990	0.193	1.000
GGT	87.03 ± 30.11	78.85 ± 30.29	75.54 ± 18.22	82.25 ± 35.39	59.79 ± 13.70 cc	90.36 ± 19.96 ab	92.59 ± 22.20 a	0.447	<0.0001	0.857
Cholesterol	62.75 ± 13.25	58.53 ± 15.05	53.90 ± 11.54	60.29 ± 14.47	49.87 ± 9.12	64.35 ± 10.19 ab	62.38 ± 11.56 ab	0.124	<0.0001	0.114

SOD, superoxide dismutase; GSH-px, glutathione peroxidase; H_2_O_2_, hydrogen peroxide; ALT, alanine aminotransferase; AST, aspartate aminotransferase; ALP, alkaline phosphatase; GGT, gamma-glutamyl transferase. Control: without additives; FO: functional oils, 0.50 g/kg DM of castor and cashew nut shell oils; SeE: hydroxy-selenomethionine and vitamin E, 0.50 mg/kg of selenium and 100 IU/kg DM of vitamin E; FO plus SeE, at the same doses as the other groups. Means in the same row followed by different lowercase letters differ significantly at the 5% level.

**Table 5:** Meat color, lipid peroxidation (TBARS) and microbiological quality as measures of the shelf life of meat (means ± s.d.) of Dorper x Santa Ines lambs fed a high-concentrate diet with different antioxidants.

Measurements	Treatment				Days on Display			*p*-Value		
	Control	FO	FO Plus SeE	SeE	0	3	5	Treatment	Time	Treatment * Time
Color L*	42.18 ± 3.74 c	44.02 ± 3.74 ab	44.79 ± 2.60 ab	44.84 ± 2.60 ab	42.56 ± 3.81 bc	45.40 ± 4.17 a	43.91 ± 2.04 bc	0.026	0.005	0.630
Color a*	14.87 ± 4.49	14.29 ± 3.76	13.84 ± 3.64	14.09 ± 3.78	15.43 ± 2.70	13.76 ± 4.50	13.63 ± 1.79	0.830	0.140	0.999
Color b*	11.87 ± 2.08	12.99 ± 2.30	12.62 ± 1.09	13.11 ± 1.74	11.14 ± 3.14 c	13.41 ± 3.07 ab	13.38 ± 1.58 ab	0.121	<0.0001	0.462
TBARS ^1^	0.142 ± 0.09 a	0.197 ± 0.015 a	0.072 ± 0.04 b	0.054 ± 0.03 b	0.104 ± 0.05	0.079 ± 0.05	0.166 ± 0.13	0.001	0.020	0.401
Mesophilic	6.31 ±0.39	5.71 ± 0.33	5.58 ± 0.32	5.50 ± 0.20	2.07 ± 0.74	5.06 ± 0.38	6.37 ± 0.35	0.443	0.081	0.573
Psychrotrophic	7.74 ± 0.30	7.72 ± 0.46	7.23 ± 0.19	7.14 ± 0.18	2.06 ± 0.20	6.11 ± 0.17	8.01 ± 0.84	0.056	<0.0001	0.021
*Staphylococcus* spp.	6.51 ± 0.37	5.11 ± 0.24	4.05 ± 0.15	5.74 ± 0.11	2.39 ± 0.27	3.22 ± 0.20	6.47 ± 0.39	0.061	0.010	0.007
Enterobacteriaceae	6.38 ± 0.33	5.39 ± 0.42	4.43 ± 0.40	5.83 ± 0.12	2.03 ± 0.18	4.38 ± 0.36	6.40 ± 0.37	0.309	0.016	0.050

Means in the same row followed by different lowercase letters differ significantly at the 5% level. ^1^ Thiobarbituric acid reactive substances (μg malondialdehyde/kg tissue). The color measurements were made with a portable spectrophotometer operating in the CIE L*a*b system, where L* is the chroma associated with brightness (L * = 0 black, 100 white), a* is the chroma that varies from green to red, and b* is the chroma that varies from blue to yellow (CIE, 1986). Control: without additives; FO: functional oils, 0.50 g/kg DM of castor and cashew nut shell oils; SeE: hydroxy-selenomethionine and vitamin E, 0.50 mg/kg of selenium and 100 IU/kg DM of vitamin E; FO plus SeE, at the same doses as the other groups.
